# Prospective Registry of Outcomes, Treatment, and Clinical Trajectories for Anti–IFN-γ Immunodeficiency

**DOI:** 10.1001/jamanetworkopen.2026.23220

**Published:** 2026-07-15

**Authors:** Valerie Chiang, Freya Kit Lam Chung, Nga Yi Leung, Wai Ki Ip, Gordon Kwok Ho Chu, James Kwong Yew Hooi, Jane Chi Yan Wong, Elaine Yuen Ling Au, Philip Hei Li

**Affiliations:** 1 Division of Clinical Immunology, Department of Pathology, Queen Mary Hospital, Hong Kong SAR, China; 2 Division of Rheumatology and Clinical Immunology, Department of Medicine, Queen Mary Hospital, The University of Hong Kong, Hong Kong SAR. China

## Abstract

**Importance:**

Autoantibodies against interferon-γ (AIGA) impair the IFN-γ–interleukin 12 pathway. The disease is underrecognized, leading to profound diagnostic and therapeutic delays. Evidence on treatment strategies, particularly B-cell depletion, remains limited.

**Objective:**

To evaluate the utility of Stat1 phosphorylation (pStat1) neutralization assays to guide management of AIGA immunodeficiency.

**Design, Setting, and Participants:**

This ambispective longitudinal cohort study of patients with confirmed AIGA immunodeficiency was performed at Queen Mary Hospital, as the only center performing AIGA testing under Hong Kong’s unified public health care system. The AIGA Prospective Registry of Outcomes, Treatment and Evaluating Clinical Trajectories (PROTECT) reviews patients with AIGA immunodeficiency in Hong Kong, with a focus on rituximab response. Participants included patients with AIGA immunodeficiency demonstrated by positive enzyme-linked immunosorbent assay findings with pStat1 inhibition recruited into the AIGA-PROTECT registry. Demographic, clinical, and laboratory data were collected longitudinally from January 1, 2021, to November 30, 2024.

**Exposures:**

All patients diagnosed with AIGA immunodeficiency were evaluated for rituximab therapy by the attending immunologist on a case-by-case basis. Treatment decisions were guided by clinical severity, infection burden, and patient preference, following detailed counselling on potential benefits and risks.

**Main Outcomes and Measures:**

Demographic characteristics, medical history (with detailed documentation of prior and incident hospitalizations), physical examination findings, laboratory investigations including serial pStat1 neutralizing assays, and longitudinal clinical progress were collected. For patients treated with rituximab, their laboratory and clinical parameters before and after treatment were compared.

**Results:**

A total of 38 patients with AIGA immunodeficiency were recruited. Patients experienced disseminated infections from multiple pathogens. Twenty-one patients (55.3%) received rituximab, which was associated with reduced patient hospitalizations (odds ratio, 25.00 [95% CI, 1.48-422.26]; *P* < .001) and annual hospitalization rates (median difference, −3.0 [95% CI, −5.0 to −1.0]; *P* = .008). Serial monitoring of pStat1 neutralization aligned with clinical events.

**Conclusions and Relevance:**

This cohort study presents a detailed analysis of clinical features, course of disease, and outcomes of rituximab treatment in patients with AIGA immunodeficiency in a clinical setting. These findings suggest that serial monitoring via pStat1 assays is a valuable tool for monitoring treatment response and guiding clinical management.

## Key Points

**Question:**

What are the clinical features and management strategies for immunodeficiency associated with anti–interferon-γ autoantibodies (AIGA)?

**Findings:**

This cohort study of 38 patients with AIGA immunodeficiency, including 21 treated with rituximab in a clinical setting, found sustained infection remission, improved survival, and acceptable safety profiles.

**Meaning:**

Results of this study suggest that this rare immunodeficiency syndrome can be effectively managed with rituximab and monitored by functional Stat1 phosphorylation neutralization assays.

## Teaser

This cohort study assesses the outcomes of rituximab-treated patients with immunodeficiency associated with anti–interferon-γ autoantibodies for as long as 4 years of posttreatment follow-up.

## Introduction

Autoantibodies against cytokines lead to acquired immunodeficiency syndromes, typically with adult onset. The clinical features are phenotypically similar to those of diseases caused by monogenic defects in related receptors, or proteins involved in signaling pathways.^1^ As such, the International Union of Immunological Societies Primary Immunodeficiency Expert Committee classifies anti–cytokine autoantibodies as “phenocopies of primary immunodeficiencies.”^2^ In particular, immunodeficiency characterized by anti–interferon-γ autoantibodies (AIGA) results in a syndrome that mimics many characteristics of mendelian susceptibility to mycobacterial disease, with increased susceptibility to nontuberculous mycobacteria (NTM) and other opportunistic intracellular pathogens.^3,4,5^ AIGA immunodeficiency neutralizes endogenous IFN-γ to impair the IFN-γ–interleukin 12 pathway, compromising macrophage-mediated intracellular killing and leading to infections that are severe, disseminated, and life-threatening.^6,7,8,9^ Our study aims to characterize the clinical features and management strategies for AIGA-associated immunodeficiency.

The exact pathogenesis of AIGA immunodeficiency remains inadequately understood. It has been hypothesized that antibodies develop due to molecular mimicry against *Aspergillus* species, with specific binding regions and antigenic epitopes showing homology to the *Aspergillus* Noc2 protein. However, how this binding mechanism leads to the specific spectrum of disease and heterogenous clinical manifestations remain to be further elucidated.^10,11^ Due to the rarity of AIGA immunodeficiency, it is profoundly underdiagnosed and underappreciated. However, within the present decade, there has been growing recognition of the condition, especially in the Asia Pacific region.^5,12,13,14^

AIGAs are acquired in adulthood and patients usually present with immunodeficiency after 30 to 40 years of age. Most cases are described in East Asian ethnic groups, particularly individuals of Chinese, Thai, and Japanese descent.^3,5,12,15,16,17,18,19,20^ Significant female skewing is observed in US cohorts but is not reflected across Asia. Genetic factors are also suspected to be involved in disease susceptibility. Enrichment of HLA-DRB1*16:02 and DQB1*05:02 alleles among East and Southeast Asian cohorts has been previously reported, but suggest incomplete penetrance when examined in Western populations, which may be due to inherent limitations of comparing specific HLA alleles across different ancestry groups.^21,22,23^

Laboratory diagnosis of AIGA immunodeficiency encompasses 2 key components, including initial detection of the autoantibodies and subsequent functional confirmation of their pathogenicity. This confirmation is typically achieved by measuring the inhibition of Stat1 phosphorylation (pStat1). Because pathogenic AIGAs neutralize IFN-γ and block downstream signaling, the failure to phosphorylate Stat1 serves as a marker of their neutralizing capacity.^24^

AIGA immunodeficiency is a chronic and persistent condition that is traditionally only monitored through clinical symptoms, which often remain inconspicuous until infections are florid and disseminated. Consequently, patients frequently experience profound delays in diagnosis and treatment, exacerbated by low clinical awareness and limited access to confirmatory assays. Poor outcomes are largely attributable to delayed diagnoses, nonstandardized testing protocols, and the absence of formal management guidelines. Patients are often left with infections that are notoriously difficult to control and refractory to antimicrobials alone. Therapeutic strategies involving the use of B-cell or plasma cell depleting agents have been documented, with rituximab being the most widely used and reported agent, demonstrating clinical improvement and reductions in antibody levels.^25,26,27^ However, evidence is limited to small case series, and without formal clinical trials, the efficacy of these treatments remains to be established.

Hong Kong’s unified public health care system supports comprehensive patient identification and consistent clinical management across the territory, with the Division of Clinical Immunology at Queen Mary Hospital serving as the sole reference laboratory for diagnosis of AIGA immunodeficiency. This centralized platform enables standardized testing and systematic collection of clinical and immunological follow-up data of all confirmed cases in the population. Leveraging this infrastructure, we established the AIGA Prospective Registry of Outcomes, Treatment and Evaluating Clinical Trajectories (AIGA-PROTECT), a prospective longitudinal cohort study designed to define the natural history, treatment response, and useful biomarkers in AIGA immunodeficiency, including serial pStat1 assessments. This report presents the largest clinical experience, to our knowledge, of rituximab-treated patients with AIGA immunodeficiency with as long as 4 years of posttreatment follow-up.

## Methods

### Study Design and Cohort

We conducted an ambispective longitudinal cohort study at Queen Mary Hospital, the sole tertiary referral center in Hong Kong providing diagnostic testing for AIGA. All patients with laboratory-confirmed AIGA immunodeficiency, demonstrated by positive enzyme-linked immunoassay (ELISA) findings and confirmed functional neutralization of Stat1, were prospectively enrolled at diagnosis between January 1, 2021, and November 30, 2024, into the AIGA-PROTECT registry. Retrospective clinical data prior to 2021 were retrieved from Hong Kong’s unified electronic patient records system. As the only center performing AIGA testing under Hong Kong’s unified public health care system, this registry captures the entire referred population with AIGA immunodeficiency in the territory during the study period. This cohort study was conducted and reported in accordance with the Strengthening the Reporting of Observational Studies in Epidemiology (STROBE) guidelines. The study was approved by the Institutional Review Board of the University of Hong Kong and the Hospital Authority Hong Kong West Cluster. Informed consent was waived due to the retrospective design of the study and the use of anonymized data.

Comprehensive baseline data were collected, including demographic characteristics, medical history (with detailed documentation of prior and incident hospitalizations), physical examination findings, laboratory investigations, and longitudinal clinical progress through scheduled follow-up visits. The onset of disease was defined by the occurrence of a salient infection, namely any infective symptoms that were clinically attributable to AIGA immunodeficiency.

### Rituximab Treatment

In Hong Kong, AIGA immunodeficiency is not a listed indication for rituximab, so it is used as an off-label self-financed item. All patients diagnosed with AIGA immunodeficiency were evaluated for rituximab by the attending immunologist on a case-by-case basis. Treatment decisions were guided by clinical severity, infection burden, and patient preference, following detailed counselling on potential benefits and risks. After the indications and potential adverse effects were explained, consenting patients proceeded with rituximab therapy. The regimen consisted of two 1000-mg intravenous doses administered 2 weeks apart, with repeat courses as deemed clinically indicated.

### Laboratory Assessment

Serum samples were collected at serial clinical time points between January 1, 2021, and November 30, 2024. Samples were tested with at least 1 of the following methods adapted from literature^28,29^: ELISA for AIGA, and assessment of pStat1 inhibition by AIGA. In our study, we used a functional neutralization assay wherein healthy donor monocytes were stimulated with IFN-γ in the presence of patient serum, including undiluted serum samples and serial dilutions of 1:10, 1:100, 1:1000, and 1:10 000 (eFigure 1 and eFigure 2 in Supplement 1). The final neutralizing titer was defined as the highest serum dilution that inhibited pStat1. Higher dilutions still producing inhibition imply stronger antibody activity. Detailed descriptions of these methods and representative graphics are provided in eMethods 1 in the Supplement 1.

### Statistical Analysis

Categorical variables were expressed as numbers (percentages), and continuous variables were expressed as medians (ranges). Descriptive analysis with literature review was conducted for clinical characteristics. Comparison was also performed between patients before and after treatment with rituximab. Laboratory parameters were compared using paired *t* tests. Clinical parameters were compared using the McNemar test for number of patients hospitalized and summarized as a matched odds ratio with 95% CI. Annual hospitalization rates were compared using the Wilcoxon signed-rank test and summarized using the Hodges-Lehmann estimate of the paired difference with 95% CIs. All statistical tests were 2 sided, and *P* < .05 was considered statistically significant. These analyses were performed using GraphPad Prism software, version 8 (GraphPad Software) and R, version 4.4.3 (R Program for Statistical Computing).

## Results

### Demographic Characteristics and Highest Reported Regional Prevalence

A total of 38 patients with positive findings for AIGA immunodeficiency were included in the analysis, including 15 females (39.5%) females and 23 males (60.5%) and median age at onset of 56 (IQR, 29-74) years. All patients were of Han Chinese ethnicity. The mean diagnostic delay was 1.7 (range, 0-10.0) years. Among those treated with rituximab, the mean duration from diagnosis to treatment initiation was 2.8 (range, 0.1-14.3) years. Mean follow-up duration was 25 (range, 0-58) months. One patient was lost to follow-up due to emigration 2 months following diagnosis. At the time of analysis, Hong Kong demonstrated the highest regional prevalence of AIGA immunodeficiency among all major published cohorts globally. Compared with these cohorts, our population had a lower proportion of female patients and an older age at clinical presentation (eTable 1 in Supplement 1).

Three patients (7.9%) had a history of cancer diagnosed either prior to or during the study period, including 1 case of renal cell carcinoma and 1 case of carcinoma of the breast that occurred prior to AIGA immunodeficiency diagnosis and 1 case of multiple myeloma that occurred after diagnosis. Seven patients (18.4%) had a documented history of autoimmunity, including Graves disease, hypothyroidism, IgG4-related disease, Sjögren syndrome, and mixed connective tissue disease, which all preceded diagnosis of AIGA immunodeficiency.

### Disseminated Polymicrobial Infections Involving Multiple Organ Systems

**Figure 1.**
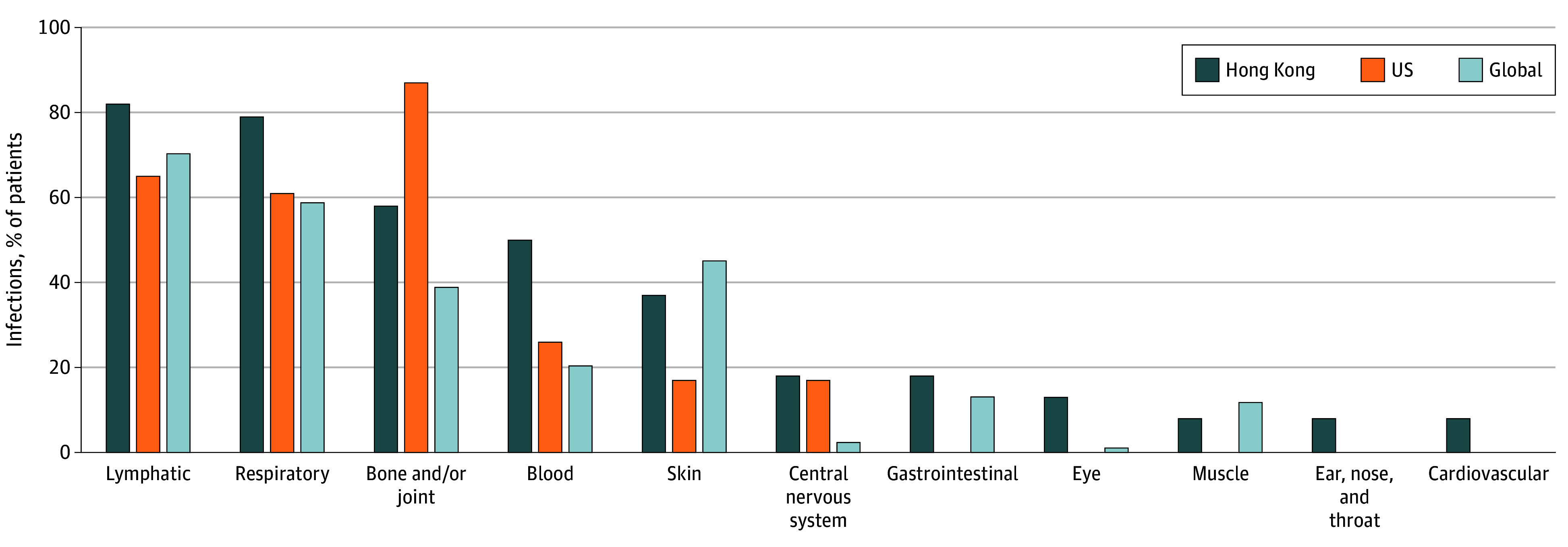
Bar Graphs of System Involvement Among Patients With Confirmed Anti–Interferon-γ Autoantibody Immunodeficiency Compared With Published Cohorts US statistics are from Hong et al^19^; global statistics, from Chen et al.^30^ Involvement of the gastrointestinal, eye, cardiovascular, and ear, nose, and throat systems were not independently reported.

Thirty-six patients (94.7%) experienced disseminated infections involving 2 or more organ systems. The lymphatic system was the most affected, followed by respiratory, bone and/or joint, blood, skin, central nervous system, gastrointestinal (including hepatic involvement), and ocular systems (Figure 1). Thirty-six patients (94.7%) required hospitalization, and 12 (31.6%) required intensive care. During the study period, 5 patients (13.2%) died due to severe infection. These deaths were attributed to *Legionella pneumophila*, *Burkholderia pseudomallei*, *Mycobacterium kansasii*, and *Mycobacterium parascrofulaceum* with *Klebsiella pneumoniae*, as well as *Salmonella* species with *Mycobacterium avium* complex (MAC).

Twenty-nine patients (76.3%) had more than 1 pathogen isolated during a single episode of infection. NTM was predominantly identified, of which MAC was most reported, followed by *M kansasii* (Table 1). Other prevalent pathogens included *Salmonella* species, *Talaromyces (Penicillium) marneffei*, and varicella zoster virus. None of the patients had concomitant *Mycobacterium tuberculosis* infections, although 5 (13.2%) reported a history of pulmonary tuberculosis.

**Table 1.** Pathogens Isolated From Patients With Confirmed Anti–Interferon-γ Autoantibody Immunodeficiency

Pathogen	Patients affected, No. (%)	Sites of positive isolation
*Mycobacteria*	34 (89.5)	NA
Slow growing		
*Mycobacterium avium* complex	16 (42.1)	Lymph node, spleen, respiratory (sputum, BAL, pleural fluid); blood, vessel wall; bone (bone biopsy, abscess pus); skin; muscle (abscess pus); ENT (nasopharyngeal biopsy)
* Mycobacterium kansasii*	10 (26.3)	Lymph node; respiratory (sputum, BAL); blood; skin
* Mycobacterium parascrofulaceum*	2 (5.3)	Lymph node; respiratory (tracheal aspirate); blood; skin (tissue, abscess pus)
* Mycobacterium paragordonae*	2 (5.3)	Sputum
* Mycobacterium mageritense*	2 (5.3)	BAL
* Mycobacterium paraense*	1 (2.6)	Skin
Rapid growing		
* Mycobacterium abscessus*	7 (18.4)	Lymph node; sputum; blood
* Mycobacterium fortuitum*	4 (10.5)	Lymph node; sputum
* Mycobacterium chelonae*	3 (7.9)	Sputum; skin
Bacteria	23 (60.5)	NA
*Salmonella* species, nontyphoidal	14 (36.8)	Respiratory (BAL); blood; bone and/or joint (joint fluid); GI (bile, peritoneal fluid); muscle (abscess pus)
* Legionella pneumophila*	4 (10.5)	Respiratory (sputum, pleural fluid)
* Burkholderia pseudomallei*	3 (7.9)	Blood
* Klebsiella pneumoniae*	3 (7.9)	Respiratory (sputum, BAL); bone and/or joint (joint fluid); GI (gastric aspirate)
* Pseudomonas aeruginosa*	3 (7.9)	Respiratory (BAL, tracheal aspirate); GI (bile)
Others, including *Neisseria* species, *Hemophilus* species, *Listeria monocytogenes*, *Staphylococcus aureus*, *Mycoplasma pneumoniae*, *Acinetobacter* species	9 (23.7)	Respiratory (sputum, BAL); blood; eye (corneal scraping)
Fungi	12 (31.6)	NA
* Talaromyces marneffei*	8 (21.1)	Lymph node; respiratory (sputum, lung tissue); blood; cardiovascular (pericardial fluid)
* Candida albicans*	3 (7.9)	Respiratory (sputum, BAL)
* Pneumocystis jirovecii*	1 (2.6)	BAL
Virus	11 (28.9)	NA
Varicella zoster virus	8 (21.1)	Skin (tissue, vesicular fluid); CNS (cerebrospinal fluid)
Cytomegalovirus	3 (7.9)	BAL with viremia; GI (intestinal biopsy)
Epstein-Barr virus	2 (5.3)	ENT (nasopharyngeal biopsy); skin

Abbreviations: BAL, bronchoalveolar lavage; CNS, central nervous system; ENT, ear, nose and throat; GI, gastrointestinal; NA, not applicable.

### Rituximab Treatment

**Figure 2.**
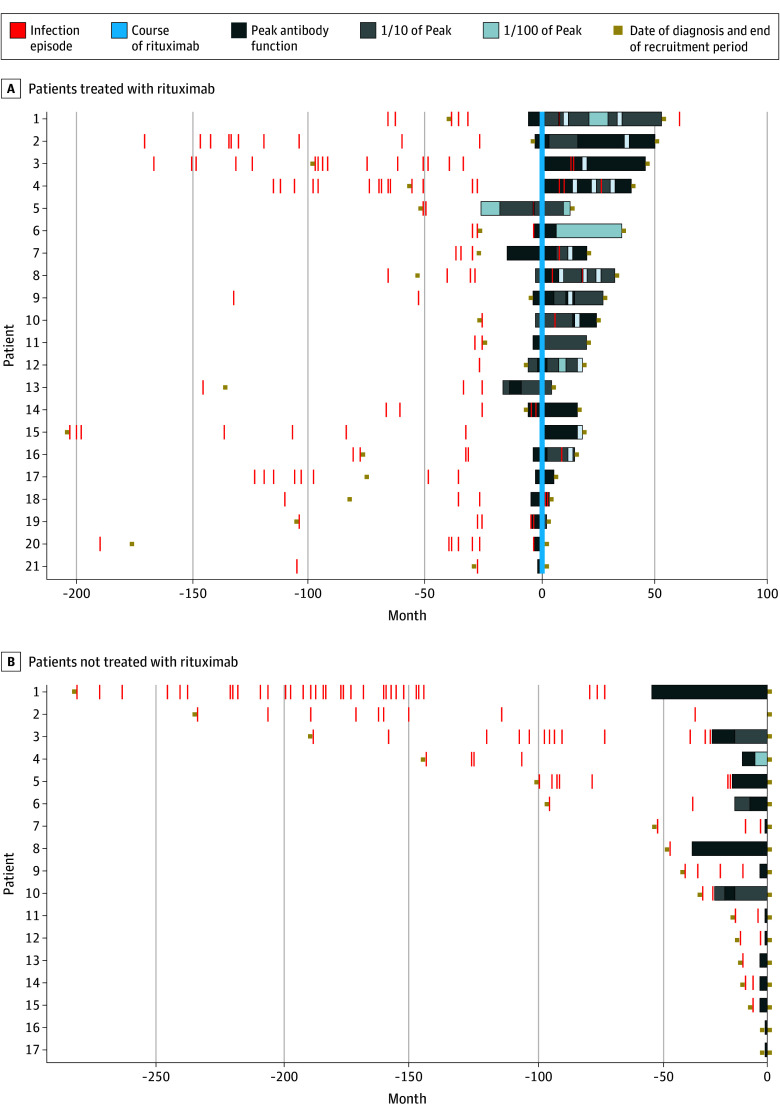
Longitudinal Timeline Plot of Course of Patients With Confirmed Anti–Interferon-γ Autoantibody Immunodeficiency The bars around 0 denote annual infection-related hospitalizations before and after first rituximab treatment. The red lines indicate infection episodes. The dots indicate date of diagnosis and end of recruitment period.

All patients were treated with antimicrobials tailored to isolated pathogens. Twenty-one patients (55.3%) received rituximab, including 5 who received rituximab from other referring physicians. Treated patients used a mean of 1.76 cycles (range, 0-4 cycles). Overall, patients treated with rituximab showed greater clinical and in vitro improvement compared with patients who used antimicrobials only (Figure 2). There were no significant differences in baseline characteristics of patients who received rituximab and those who did not (eTable 2 and eMethods 2 in Supplement 1).

In the year following the first dose of rituximab, patient hospitalizations were significantly reduced with a matched odds ratio of 25.00 (95% CI, 1.48-422.26; *P* < .001). Similarly, annual hospitalization rates per individual decreased significantly, with paired difference of −3.0 (95% CI, −5.0 to −1.0; *P* = .008) (Table 2). Reinfections, if present, occurred a mean of 9 months (range, 0-32 months) after the first course of rituximab. In contrast, there were no noticeable changes in infective events in patients who did not receive rituximab (Figure 2B). Multivariable regression analysis did not reveal other demographic or clinical factors associated with the number of patients hospitalized or with hospitalization rates (eTable 3 and eMethods 2 in Supplement 1).

**Table 2.** Laboratory Parameters and Clinical Outcomes Among All Patients Treated With Rituximab Before and After Treatment

Laboratory parameter or outcome	Rituximab-treated patients (n = 21)				*P* value
	Reference range	Before rituximab	After rituximab^a^	Mean paired difference (95% CI)	
IgG, median (range), mg/dL	819 to 1725	2185 (1213 to 4531)	1919 (1112 to 3964)	−279.8 (−629.4 to 69.8)	.11
IgA, median (range), mg/dL	70 to 386	340 (158 to 978)	352 (158 to 864)	5.4 (−43.5 to 54.3)	.82
IgM, median (range), mg/dL	55 to 307	118 (35 to 208)	100 (7 to 202)	−23.5 (−44.9 to −2.0)	.03
CD19^+^ B cells, median (range), cells/μL	160 to 708	215 (56 to 528)	44 (0 to 271)	−188.3 (−244.1 to −132.4)	<.001
CD4^+^ T cells, median (range), cells/μL	415 to 1418	807 (258 to 2119)	614 (276 to 1068)	−192.5 (−363.9 to −21.0)	.03
CD8^+^ T cells, median (range), cells/μL	292 to 1258	645 (219 to 1518)	507 (176 to 1009)	−83.3 (−283.8 to 117.3)	.39
CD16/56 natural killer cells, median (range), cells/μL	158 to 1156	750 (52 to 3084)	643 (151 to 1860)	−23.1 (−239.8 to 193.6)	.82
White blood cell count, median (range), cells/μL	3700 to 9200	9990 (5500 to 24 300)	8310 (4250 to 27 700)	−1700 (−4590 to 1190)	.23
Lymphocyte count, median (range), cells/μL	800 to 4800	2500 (1400 to 5370)	1990 (880 to 5230)	−450 (−770 to −130)	.008
Monocyte count, median (range), cells/μL	100 to 800	550 (280 to 1200)	1100 (90 to 10 500)	540 (−540 to 1610)	.31
Patients hospitalized, No. (%), effect estimate	NA	17 (81.0)	5 (23.8)^b^	25.0 (1.48 to 422.26)^c^	<.001
Hospitalizations per year, median (range)	NA	2 (0 to 8)	0 (0 to 9)	−3.0 (−5.0 to −1.0)^d^	.008

SI conversion factors: to convert IgA, IgG, and IgM to g/L, multiply by 0.01; lymphocyte, monocyte, and white blood cell counts to 10^9^/L, multiply by 0.001.

^a^ Unless indicated, 1 month after treatment.

^b^ Measured 12 months after treatment.

^c^ Matched odds ratio.

^d^ Hodges-Lehmann estimate of the paired difference.

Immunophenotyping performed 1 month after rituximab treatment showed expected reductions in levels of IgM, CD19^+^ B cells, and CD4^+^ T cells and absolute lymphocyte counts. No significant changes were observed in levels of IgG, IgA, CD8^+^ T cells, or natural killer cells, monocyte counts, or total white blood cell counts (Table 2). No patients required immunoglobulin replacement therapy during the entire study period.

### Serial Monitoring of pStat1 Inhibition

The absolute degree of pStat1 inhibition did not correlate with specific organ system involvement, number of systems involved, nor infection severity. However, longitudinal changes in pStat1 inhibition within individual patients correlated with clinical events. In most patients, pStat1 inhibition weakened within 4 months following rituximab treatment (patients 1, 2, 6, 7, 8, 9, 11, 12, and 16). Conversely, a rise in functional pStat1 inhibition preceded clinical relapse or reinfections (Figure 2).

### Longitudinal Timelines of Clinical Courses and Serial Laboratory Values

**Figure 3.**
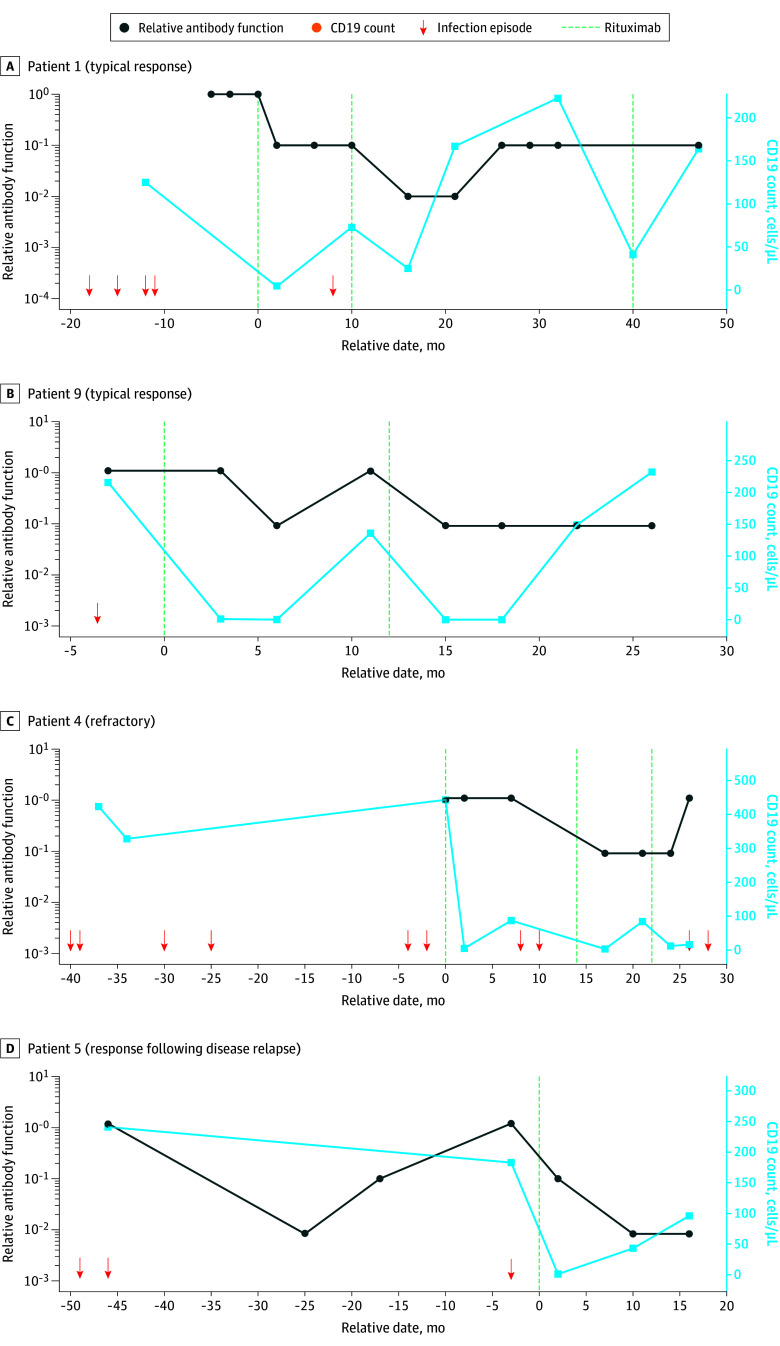
Line Graphs of Individual Clinical Courses and Serial Laboratory Markers in Selected Patients With Anti–Interferon-γ Autoantibody Immunodeficiency

Patients 1 and 9 were representative of typical response to rituximab. Both showed reductions in antibody function following the first cycle of rituximab. Patient 1 experienced 1 episode of pulmonary infection before the second cycle, while patient 9 did not. There was sustained suppression of antibody function after 2 cycles. Clinically, both patients remain infection free, despite CD19^+^ B-cell repletion (Figure 3A and B).

Patient 4 illustrates a case refractory to rituximab. The patient experienced recurrence of infections even after 4 cycles of rituximab treatment. Along with infections, the patient showed persistent elevated antibody function, despite depletion of B cells (Figure 3C).

Patient 5 illustrates the natural wax-and-wane pattern of antibodies. The patient initially presented with severe infection and septic shock. This was followed by 2 years of remission after the use of antimicrobials only, with loss of antibody function. However, a progressive increase in antibody function was observed, preceding another episode of severe infection. The patient responded to 1 cycle of rituximab, reflected by suppression of antibody function and remained infection free thereafter (Figure 3D).

## Discussion

AIGA immunodeficiency is a rare disease associated with difficulties in diagnosis and management, resulting in poor outcomes and refractory disease. The chronic nature of AIGA immunodeficiency is compounded with its fluctuating clinical manifestations, likely due to changes either in levels or functional pathogenicity of the antibodies. AIGA-PROTECT addresses critical gaps in this disease, by providing the first, to our knowledge, prospective longitudinal characterization of this syndrome and treatment outcomes in a clinical setting. We propose a shift toward proactive rituximab administration supported by a biomarker-guided management strategy.

All patients in our Hong Kong cohort were Han Chinese, and Hong Kong showed the highest prevalence among all major published cohorts. This may be due to the small geographical size of the city and the role of our center as the sole referral laboratory performing testing for AIGA, enabling near-complete case ascertainment. As this remains a highly underdiagnosed condition, the true prevalence of disease is likely underestimated, especially in countries with multiple referral centers. The proportion of females (39.5%) markedly differs from the 91% reported in the US and is slightly lower than the global estimate of 50%.^31,32^ This discrepancy suggests that female susceptibility may not be as pronounced in Asian localities and warrants further investigation.

Patients commonly present with multisystem infections, reflecting the ubiquitous role of IFN-γ in host defense. The pattern of organ system involvement and infective pathogens agrees with other studies, where multiorgan dissemination is consistently reported. The lymphatic system was the most affected, followed by respiratory, bone and/or joint, blood, and skin.^3,6,19,26^ Bacteremia was more frequent in our cohort compared with the US, where bone infections predominated and skin involvement was less common.^19^ These regional discrepancies are postulated to reflect differences in pathogen exposure.^19^ Variabilities in involvement of the central nervous system, gastrointestinal tract, and eye are likely due to underreporting rather than genuine geographical differences. More causal studies are needed to elucidate the influence of specific environmental or genetic factors on disease phenotypes.

The spectrum of pathogens in our cohort further confirms the AIGA phenotype, aligning closely with published findings, with high rates of polymicrobial infections (76.3% of episodes) and NTM predominance. In particular, MAC was the most common pathogen, but we observed relatively fewer cases of *M abscessus*, and instead more infections with *M kansasii*.^17,19^ The absence of *M tuberculosis* coinfections was consistent with prior reports, despite regional endemicity.^17^ Infections with *Cryptococcus* species are also frequently reported in literature, but none were seen in our cohort.^5,15,19,33^ In contrast, the elevated prevalence of *T marneffei* compared with overseas cohorts may be accounted for as the pathogen is endemic to Hong Kong and Southeast Asia.^6,34^ Nonetheless, our 13.2% mortality rate highlights the lethal potential of these severe infections.^30,35^

Screening for AIGA immunodeficiency is typically performed by various ligand-binding assays. ELISAs and similar enzyme immunoassays are most commonly used. Immunoblots and Western blotting also provide qualitative confirmation. Particle-based or multiplex bead arrays offer advantages in sensitivity and ability to detect multiple anti–cytokine antibodies simultaneously. In resource-limited settings, modified IFN-γ release assays such as the QuantiFERON-TB Gold In-Tube (QIAGEN) have been proposed as an initial screening tools, as they may indicate AIGA-impaired IFN-γ signaling. The use of an indeterminate QuantiFERON ELISA result has also been suggested for screening.^20,36,37^ In Hong Kong, QuantiFERON testing is self-financed and not universally available, and therefore was not used in this present study. To improve accessibility of testing, dried blood spot collection protocols have also been published.^38^ Due to a lack of commercially available assays and reference materials, tests for AIGA are generally conducted in-house at specialized laboratories and research institutions. This leads to significant heterogeneity in methods and interpretations of results. Consequently, absolute antibody titers are often unreliable for cross-patient and/or cross-cohort comparisons. There is also conflicting evidence on whether antibody titers alone are associated with disease activity.

Functional assays confirm the neutralizing capacity of AIGA on pStat1 by flow cytometry.^11,28,29^ While absolute neutralizing titers varied widely between patients and did not correlate with disease severity, within individual patients, relative changes in titers proved highly informative. We observed that a decline in pStat1 inhibition following rituximab therapy, evident within 4 months in most responders, was associated with clinical improvement, while a rising titer often preceded clinical relapse. This establishes the functional pStat1 assay not merely as a diagnostic tool, but as a dynamic biomarker for guiding pre-emptive therapy, despite its current labor-intensive nature.

Various immunomodulatory therapies for AIGA immunodeficiency have been explored and reported, including cyclophosphamide, daratumumab, bortezomib, and rituximab. Alternatives to rituximab were often used for cases unsuccessfully controlled by rituximab alone.^25,27,39,40,41,42^ A total of 21 patients (55.3%) were treated with rituximab in our cohort. Patients were given rituximab following the approved dosing for rheumatoid arthritis (1000 mg for 2 doses, 2 weeks apart). Additional doses of rituximab were given for persistent and/or recurrent infections, at a minimum of 6-month intervals (Figure 2A).

In our cohort, rituximab significantly reduced both the numbers of hospitalized patients and annual hospitalization rates. Patients treated with rituximab had fewer persistent or recurrent reinfections than those treated with antimicrobials alone, confirming the therapeutic potential of rituximab and corroborating with smaller reports.^6,25,41,43^ Treatment was well tolerated, with no adverse events observed (including infusion reactions, worsened infections, or secondary immunodeficiency). This phenomenon is likely driven by the persistence of long-lived antibody-producing cells in immune-privileged sites,^25^ which underscores the chronic nature of AIGA immunodeficiency and the necessity for lifelong monitoring.

Although previous observations suggest that patients with AIGA immunodeficiency can undergo complete remission without rituximab or other biologic treatments, we hypothesize that this stems from insufficient follow-up periods rather than true permanent recovery.^17,18,44^ While patients tend to be able to clear infections and achieve temporary remission without biologics, patient 5 illustrates the waxing-and-waning pattern of disease and likely persistence recurrence of infections (Figure 3D).

Rituximab treatment is not always successful in effectively controlling disease. Patients with AIGA immunodeficiency refractory to rituximab treatment (exemplified by patient 4) showed persistently high pStat1 neutralization function and active infections despite depletion of B cells (Figure 3C). Refractory cases previously reported also show persistently high antibody titers after rituximab treatment, but with considerable variability between individuals, making it challenging to use titers alone for prognostication across patients.^25^ This highlights the utility of functional monitoring in cases of treatment failures. For rituximab-resistant cases, alternative therapies targeting plasma cells such as bortezomib and daratumumab show promise.^27,40^

Presently, no standardized guidelines exist for rituximab dosing for AIGA immunodeficiency. As evidenced by our study, individual patients respond differently to treatment, with some requiring multiple courses to remain infection free and others unable to ever achieve disease remission despite repeated doses. This corroborates with the mixed outcomes of rituximab use seen in literature.^5,25,26,45^ We propose that serial laboratory testing, including pStat1 neutralization assays, may guide the optimal timing of rituximab administration.

### Limitations

Limitations to our study include a limited samples size, given the rarity of the disease. Episodes of infection were documented as new onset of clinical symptoms. As part of the nature of this disease, ongoing subclinical infections may have been missed. Social history and potential associated confounders were not fully available for analysis. The length of time between initiation of antimicrobial therapy and use of rituximab was not included, as treatment was often started in private care with limited data available. As rituximab treatment was self-financed, socioeconomic factors may have affected treatment availability, and dosing and confounded clinical outcomes. There were no data on patients who used more than 4 cycles of rituximab, highlighting the need for long-term studies to define optimal retreatment schedules and monitor for late effects such as secondary immunodeficiency. Finally, our laboratory assays were performed in-house; the broader application of our approach depends on the future development of commercial kits and standardized protocols to improve accessibility and reproducibility.

## Conclusions

In this ambispective cohort study of 38 patients with AIGA immunodeficiency, we presented a comprehensive analysis of clinical features, course of disease, and outcomes of rituximab treatment in a clinical setting in these patients. We included one of the largest cohorts of patients with AIGA immunodeficiency treated with rituximab and as many as 4 years of posttreatment longitudinal follow-up. Our findings establish rituximab as a cornerstone of therapy and validate serial pStat1 neutralization monitoring as a critical biomarker for guiding clinical management. We advocate for a paradigm shift away from reactive infection control toward proactive, biomarker-guided immunomodulation, to improve long-term outcomes in this challenging immunodeficiency syndrome.

## Notes

Supplement 1. **eMethods 1.** Detailed Description of Laboratory Methods **eFigure 1.** pSTAT1 at Serial Dilutions of Patient Serum Samples **eFigure 2.** Serial Dilutions of Patient Serum Incubated With Donor Monocytes Show Decreasing Severity of pSTAT1 Inhibition **eTable 1.** Baseline Characteristics of Patients With Confirmed AIGA Compared With Published Cohorts **eTable 2.** Baseline Characteristics of Patients With Confirmed AIGA According to Rituximab Treatment Status **eTable 3.** Multivariable Regression Analysis of Hospitalization Outcomes During Follow-Up in Patients With Confirmed AIGA (N = 38) **eMethods 2.** Description of Statistical Analysis Methods for eTables 2 and 3

Supplement 2.
Data Sharing Statement
