# Supplementary material for: Prospective Registry of Outcomes, Treatment, and Clinical Trajectories for Anti–IFN-γ Immunodeficiency
Source: JAMA Netw Open. 2026 Jul 15;9(7):e2623220. doi: 10.1001/jamanetworkopen.2026.23220 (PMC13373672; doi:10.1001/jamanetworkopen.2026.23220)
Supplement: Supplement 1. — eMethods 1. Detailed Description of Laboratory Methods eFigure 1. pSTAT1 at Serial Dilutions of Patient Serum Samples eFigure 2. Serial Dilutions of Patient Serum Incubated With Donor Monocytes Show Decreasing Severity of pSTAT1 Inhibition eTable 1. Baseline Characteristics of Patients With Confirmed AIGA Compared With Published Cohorts eTable 2. Baseline Characteristics of Patients With Confirmed AIGA According to Rituximab Treatment Status eTable 3. Multivariable Regression Analysis of Hospitalization Outcomes During Follow-Up in Patients With Confirmed AIGA (N = 38) eMethods 2. Description of Statistical Analysis Methods for eTables 2 and 3 [file jamanetwopen-e2623220-s001.pdf]

Supplementary Online Content

Chiang V, Chung FKL, Leung NY, et al. Prospective registry of outcomes, treatment and clinical trajectories for anti–IFN-γ autoantibodies. *JAMA Netw Open.* 2026;9(7):e2623220. doi:10.1001/jamanetworkopen.2026.23220

eMethods 1. Detailed Description of Laboratory Methods

eFigure 1. pSTAT1 at Serial Dilutions of Patient Serum Samples

eFigure 2. Serial Dilutions of Patient Serum Incubated With Donor Monocytes Show Decreasing Severity of pSTAT1 Inhibition

eTable 1. Baseline Characteristics of Patients With Confirmed AIGA Compared With Published Cohorts

eTable 2. Baseline Characteristics of Patients With Confirmed AIGA According to Rituximab Treatment Status

eTable 3. Multivariable Regression Analysis of Hospitalization Outcomes During Follow-Up in Patients With Confirmed AIGA (N = 38)

eMethods 2. Description of Statistical Analysis Methods for eTables 2 and 3

This supplementary material has been provided by the authors to give readers additional information about their work.

© 2026 Chiang V et al. *JAMA Network Open*.

eMethods 1. Detailed Description of Laboratory Methods

Enzyme-linked immunoassay (ELISA) for detection of AIGA

Microplates were coated with recombinant human IFNγ. Stock recombinant human IFNγ (BD Pharmingen, Cat. No. 554617) was diluted to 1:100 with coating buffer (bicarbonate buffer of pH 9.6) to yield a concentration of 1ug/mL recombinant IFNγ. 100µl of the diluted solution was added to each well of a Nunc-Immunoplates (Thermo Scentific, Cat. No. 439454) 96-well ELISA plate and incubated at 4°C overnight and blocked with blocking solution (2% BSA with phosphate-buffered solution (PBS)).

Patients' sera were diluted 1:500 with the blocking solution, and 100µl of the diluted samples were added to each well. Each ELISA plate included two blank wells, a positive control (in-house), a negative control (in-house), and patient samples performed in triplicate.

Plates were then incubated with an HRP-conjugated polyclonal rabbit anti-human IgG (Dako P0214), followed by a TMB chromogen substrate (Inova, Cat. No. 508504), and finally stopped with an HRP stop solution 1N H2SO4 (Inova, Cat. No. 508509)).

Optical density (OD) values were measured at 450nm with reference filter at 620 nm by an ELISA reader.
Each sample's OD index was calculated by taking the average OD of the three samples and divided by the average of the two blank samples.

Reference range

Samples with an OD index of above 0.075nm (established from the 99th percentile of 120 healthy adult controls) were considered positive.

Detection of neutralizing function of AIGA (functional assessment)

To determine the neutralizing activity of anti-IFN-g autoantibodies in patient sera on IFNγ signaling, the inhibition of IFNγ-induced STAT1 phosphorylation were analyzed.

Stimulation of donor leukocytes

45ul of heparinized whole blood obtained from healthy donors was transferred into test tubes. 5ul of anti-human CD14-FITC (BD, Cat No. 555397) was added to all tubes, except for unstained control tubes. Blood was stimulated with human recombinant IFNγ (R&D 285-IF-100) at concentrations of 100ng/mL and 50ng/mL for 15 min at 37°C water bath (except the baseline unstimulated, stained tube).

Preparation of controls

Negative control, normal control and positive control tubes were prepared by pre-incubating PBS, healthy normal serum and an anti-human IFNγ antibody Clone B27 (Immunotools, Cat No. 21853530) with 100ng/ml IFNγ respectively for 15 minutes at 37°C water bath.

© 2026 Chiang V et al. *JAMA Network Open*.

Preparation of patient samples

Tubes containing healthy donor leukocytes were prepared with PBS alone, (unstimulated), an in-house normal control sera, an in-house known positive control sera (at neat concentration), and various dilutions of patient sera (neat, 1:10, 1:100, 1:1000, and 1:10000). 4 points 10-fold serial dilution of patient sera were prepared with PBS. Sera in neat and with various dilutions were added to separate tubes of 100ng/ml IFNγ and were incubated for 15 minutes at 37°C water bath to allow neutralization, if any, to occur. The pre-treated samples were then added to donor whole blood as stimulants and tubes were incubated at 37°C water bath for 15 minutes.

Detection of STAT1 phosphorylation in leukocytes

Red cells were lysed with Whole Blood Lysing reagent (Beckmann Coulter Immunoprep) and leucocytes were fixed by TQ Prep (Beckmann Coulter). Leukocytes were then permeabilized on ice by pre chilled permeabilization buffer (BD Phosflow™ Perm Buffer III) for 30 minutes and stained by anti-pSTAT1 (pY701)-PE (BD, Cat No. 612564) for 15 minutes in the dark. After washing, cells were resuspended in PBS and analyzed by flow cytometry.

Data collection and assessment by flow cytometry

Data was collected with a FC500 flow cytometer (Beckman Coulter) and analyzed with Kaluza software (Beckman Coulter).

Data analysis

CD14+ monocytes were analyzed and pSTAT1 was evaluated. The pSTAT1-Stimulation index (SI) was calculated by dividing the mean fluorescence intensity (MFI) of sample pSTAT1+ cells over the MFI of baseline pSTAT1+ cells.

Reference range

An in-house reference range of stimulation index was established by recruiting 50 healthy normal subjects to perform STAT1 phosphorylation assay. Normal reference range in SI was 2.06 - 3.42 (calculated by mean ± 2 SD).

Determination of dilution cutoff for neutralizing activity.

Stimulation index of each dilution tube was calculated. Sample with SI lower than normal reference range was determined to possess anti-IFNγ neutralizing activity. The cutoff was set at the highest dilution which showed the neutralizing activity.

© 2026 Chiang V et al. *JAMA Network Open*.

eFigure 1. pSTAT1 at Serial Dilutions of Patient Serum Samples

© 2026 Chiang V et al. *JAMA Network Open*.

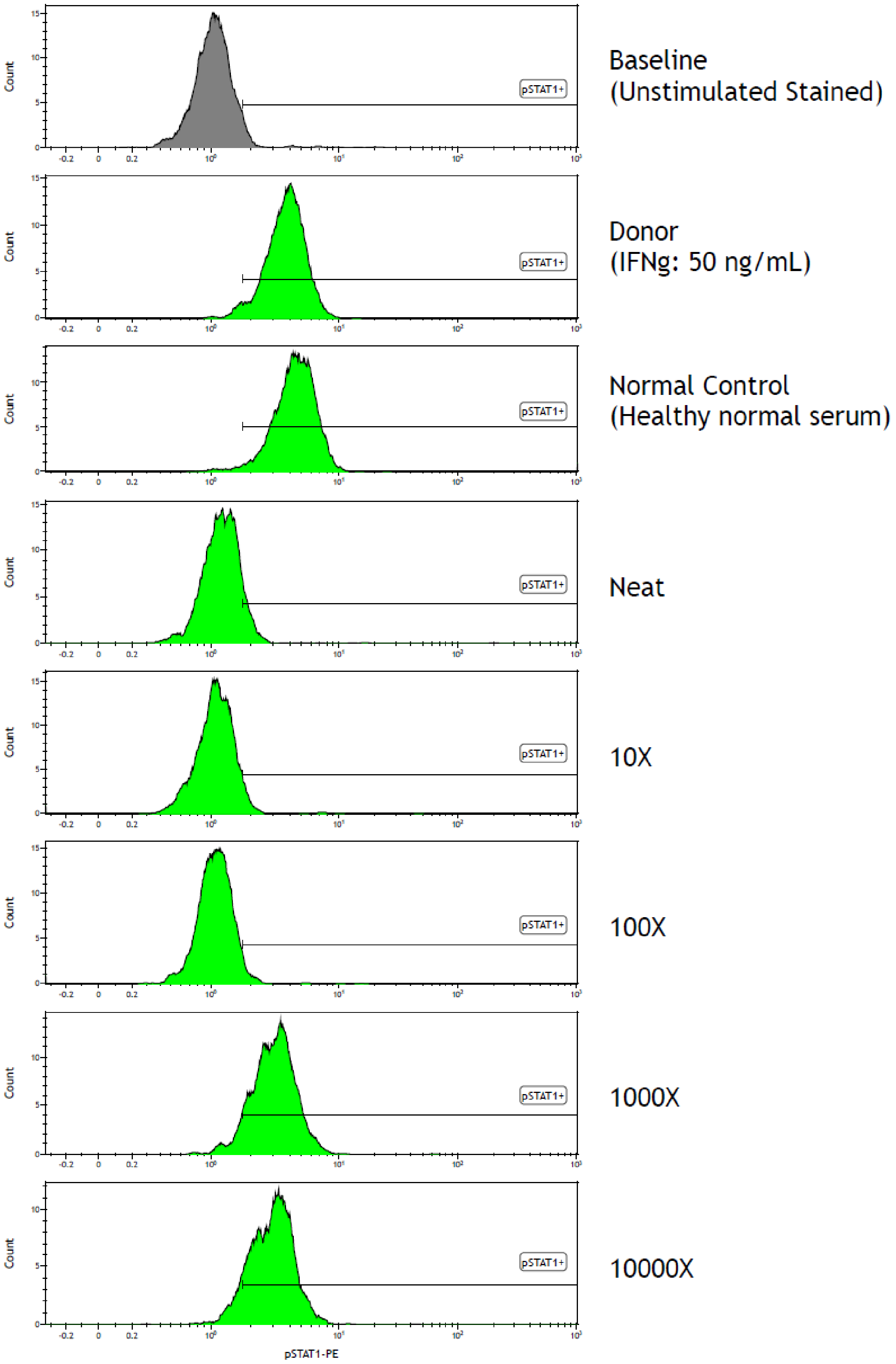

© 2026 Chiang V et al. *JAMA Network Open*.

Healthy donor monocytes were used for all samples. The unstimulated control is shown at the top of the figure (grey). Stimulation with recombinant IFNγ at 50ng/mL led to phosphorylation in a normal control and donor leukocytes.
The presence of patient plasma at serial dilutions markedly inhibited the phosphorylation of STAT1 response to IFNγ.
Both the number of cells activated and the MFI intensity of activation were markedly reduced.
Inhibition of pSTAT1 showed slight improvement with serial dilutions of patient plasma.

eFigure 2. Serial Dilutions of Patient Serum Incubated With Donor Monocytes Show Decreasing Severity of pSTAT1 Inhibition

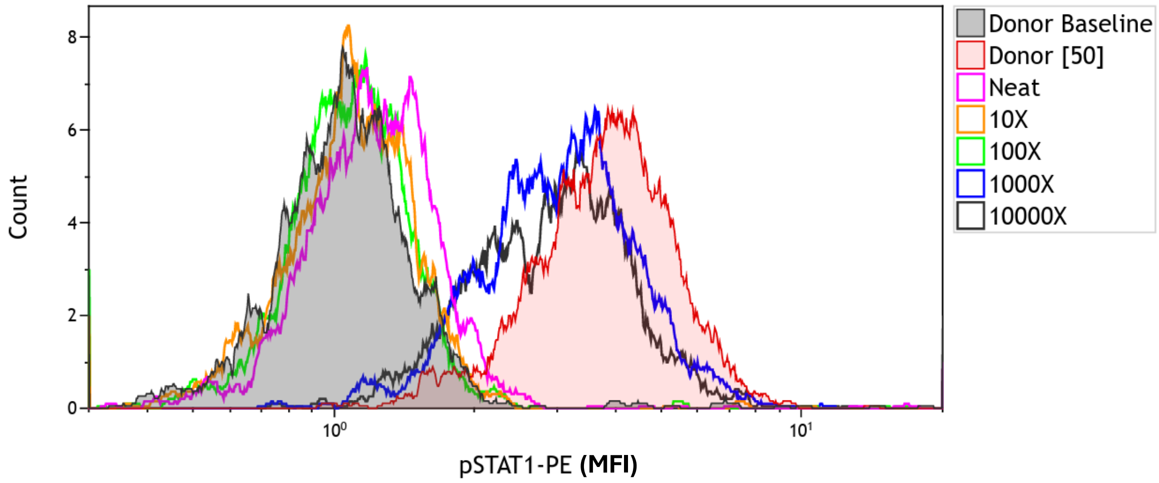

Neutralization function was reported at 1:100 for this sample.

© 2026 Chiang V et al. *JAMA Network Open*.

eTable 1. Baseline Characteristics of Patients With Confirmed AIGA Compared With Published Cohorts

	Independent Cohorts						Combined Rev[illegible]	
**Cohort**	**Hong Kong** (present study)	Thailand[15] (Angkasekwinai)	Mainland China[5] (Qiu)	Taiwan[17] (Chi)	Japan[16] (Aoki)	US[19] (Hong)	Hase[18]	Z[illegible]
**Sample size**	**38**	80	63	45	30	23	111	[illegible]
**Prevalence (per million) #**	**5.17**	1.12	1.30	1.91	0.24	0.07	NA	[illegible]
**Female, n (%)**	**15 (40%)**	41 (51%)	29 (46%)	24 (53%)	16 (53%)	21 (91%)	64 (58%)	[illegible]
**Age, median (range)**	**56 (29-74)**	50 (20-76)	53 (45-63)	56 (50-67)	66 (63–69)	45 (35-59)	52 (15-87)	[illegible]
**Deceased, n (%)**	**5 (13%)**	4 (5%)	9 (14%)	NA	NA	0	NA	[illegible]
**Delay in diagnosis (years)**	**1.7 (0-10)**	NA	NA	NA	NA	NA	NA	[illegible]
**History of tuberculosis**	**5 (13%)**	12 (13%)	NA	4 (9%)	NA	NA	NA	[illegible]
**History of malignancy*, n (%)**	**3 (7%)**	NA	NA	4 (9%)	1 (3%)	NA	NA	[illegible]
**History of autoimmunity**, n (%)**	**7 (18%)**	2, 2.5	NA	4 (9%)	NA	NA	NA	[illegible]

# Estimated prevalence was calculated based on the cohort sample size and publicly available census data on population demographics.

* Malignancy: renal cell carcinoma, carcinoma of the breast, multiple myeloma, acute promyelocytic leukemia

** Autoimmunity: Graves' disease, hypothyroidism, IgG4-related disease, Sjögren's syndrome, and mixed connective tissue disease

© 2026 Chiang V et al. *JAMA Network Open*.

eTable 2. Baseline Characteristics of Patients With Confirmed AIGA According to Rituximab Treatment Status

Variable	Rituximab-treated (n=21)	Not treated with rituximab (n=17)	p-value
Age, years	65 (45–78)	62 (31–75)	0.52
Female sex, n (%)	8 (38.1%)	7 (41.2%)	0.85
Delay in diagnosis, years	1.0 (0.0–9.5)	1.0 (0.0–2.0)	0.07
Number of organ systems involved	4 (1–7)	3 (1–6)	0.31
Previous sepsis, n (%)	9 (42.9%)	10 (58.8%)	0.33
History of tuberculosis, n (%)	3 (14.3%)	2 (11.8%)	0.82
History of malignancy, n (%)	2 (9.5%)	1 (5.9%)	0.68
History of autoimmunity, n (%)	5 (23.8%)	2 (11.8%)	0.34
Previous immunosuppression, n (%)	4 (19.0%)	0 (0.0%)	0.06
Hospitalized during baseline window, n (%)	17 (81.0%)	13 (76.5%)	0.74
Baseline hospitalization rate, per year	2.0 (0.0–8.0)	3.0 (0.0–9.0)	0.85
Baseline CD4 count, cells/µL	884 (345–1494)	628 (237–1336)	0.18

eTable 3. Multivariable Regression Analysis of Hospitalization Outcomes During Follow-Up in Patients With Confirmed AIGA (N = 38)

Variable	Hospitalized during follow-up (95% CI)	p-value	Hospitalizations during follow-up (95% CI)	p-value
Rituximab treatment	0.05 (0.01–0.34)	0.002	0.28 (0.08–0.90)	0.03
Age, years	0.98 (0.90–1.07)	0.70	0.97 (0.92–1.02)	0.19
Female	0.73 (0.12–4.46)	0.74	0.52 (0.17–1.65)	0.27
Baseline hospitalization rate, per year	0.95 (0.68–1.35)	0.76	0.98 (0.77–1.25)	0.86
Number of organ systems involved	0.83 (0.43–1.61)	0.59	0.65 (0.42–1.00)	0.052
Previous sepsis	0.69 (0.08–5.96)	0.74	1.99 (0.48–8.29)	0.34

eMethods 2. Description of Statistical Analysis Methods for eTables 2 and 3

Categorical variables were presented as numbers (percentages), and continuous variables as medians (ranges). Baseline characteristics were compared using Pearson's chi-square test or the Wilcoxon rank-sum test, as appropriate. Follow-up hospitalization outcomes were analyzed using multivariable generalized linear models. All tests were 2-sided, and $P < 0.05$ was considered statistically significant. Statistical analyses were performed using R version 4.4.3 (R Foundation for Statistical Computing, Vienna, Austria).
