# Supplementary material for: Prospective Registry of Outcomes, Treatment, and Clinical Trajectories for Anti–IFN-γ Immunodeficiency
Source: JAMA Netw Open. 2026 Jul 15;9(7):e2623220. doi: 10.1001/jamanetworkopen.2026.23220 (PMC13373672; doi:10.1001/jamanetworkopen.2026.23220)
Supplement: Supplement 2. — Data Sharing Statement [file jamanetwopen-e2623220-s002.pdf]

Data Sharing Statement

Chiang. Prospective Registry of Outcomes, Treatment, and Clinical Trajectories for Anti–IFN-γ Immunodeficiency. *JAMA Netw Open*. Published July 15, 2026.
doi:10.1001/jamanetworkopen.2026.23220

Data
Data available: No
